# A Comparison of an Alternative Weight-Grading Model Against Chronological Age Group Model for the Grouping of Schoolboy Male Rugby Players

**DOI:** 10.3389/fphys.2021.670720

**Published:** 2021-06-10

**Authors:** Grégory Lentin, Sean Cumming, Julien Piscione, Patrick Pezery, Moez Bouchouicha, José Gadea, Jean-Jacques Raymond, Pascale Duché, Olivier Gavarry

**Affiliations:** ^1^Research Unit “Impact of Physical Activity on Health” (IAPS n° 201723207F), University of Toulon, Toulon, France; ^2^Department of Health, University of Bath, Bath, United Kingdom; ^3^Department of Performance, French Rugby Federation, Marcoussis, France; ^4^Université de Toulon, Aix Marseille Univ, CNRS, LIS, Marseille, France; ^5^Ligue Sud Provence-Alpes Côte d’Azur de Rugby, Le Pradet, France; ^6^Sport Medicine and Traumatology Unit, CHITS, Toulon, France

**Keywords:** health, rugby union, schoolboy, obesity, grading

## Abstract

**Objectives:**

Concerns regarding marked differences in the weights and body composition of young rugby players competing within the same age groups have led to the suggestion of alternative models for grouping young players. The aims of this study were (1) to compare variance in the body size and body composition of schoolboy rugby players (9 to 14 years), across weight- and age-grading models, and (2) to identify morphotypes for the weight model using Hattori’s body composition chart.

**Materials and Methods:**

Skinfold thickness measurements were used to assess body fat mass (BF), fat-free mass (FFM), body fat mass index (BFMI), and fat-free mass index (FFMI). Standardized measure of height and weight were taken for all participants. Data were grouped according to the age categories of the French Rugby Federation (U11: Under 11 years, U13: Under 13 years, and U15: Under 15 years), and to the weight categories (W30–44.9; W45–59.9; and W60–79.9) carried out from 25th and 75th weight percentile in each age category. Body mass index status (NW normal-weight versus OW/OB overweight/obese) was considered. Extreme morphotypes are characterized from BFMI and FFMI in the weight-grading model on Hattori’s body composition chart.

**Results:**

The dispersion of anthropometric characteristics decreased significantly for the weight model, except for height in all groups and BFMI for U13. Among NW, 3, 1.8, and 0% upgraded; 18.2, 68.7, and 45.5% downgraded; among OW, 50, 21.5, and 12.5%; and among OB, 91.3, 83.3, and 74.6% upgraded, respectively, in U11, U13, U15. FFMI/BFMI were correlated in U11 (*r* = 0.80, *p* < 0.001), U13 (*r* = 0.66, *p* < 0.001), and U15 (*r* = 0.77, *p* < 0.001). There was no significant correlation in W45–59.9 and low correlations in W30–44.9 (*r* = 0.25, *p* < 0.001) and W60–79.9 (*r* = 0.29, *p* < 0.001). Significant grading difference between the centroids (*p* < 0.05) and the distribution deviates from centroids of BFMI and FFMI (*p* < 0.0001) were noted between the two models. Thirteen players were located in adipo-slender, twenty-three in adipo-solid, twenty-two in lean-slender, and two located in the lean-solid morphotype in weight model.

**Conclusion:**

A weight-grading model should be considered to limit mismatches in anthropometric variables. However, variations of body composition also persisted for this model. Hattori’s body composition chart allowed more detailed examination of morphological atypicalities among schoolboy rugby players.

## Introduction

In the last decades, the evolution of rugby union has moved toward a significant increase in the physical size among the players. The trend toward “heavier and taller” players is observed in adolescent rugby players (Sedeaud et al., 2013). This secular change can be explained by a number of factors, including more extensive and competitive advancements in player identification and selection and greater investment in conditioning techniques and resources for junior players, and secular increases in height and weight among the general youth population during the last decades (Bartkowiak et al., 2020). In sports such as rugby, young players can focus on the desire to gain weight during growth, especially as muscle mass, to improve strength, power, and speed performance (Carl et al., 2017). Increases in both lean and body mass could, however, enhance the impact forces experienced in the tackle (Pain et al., 2008) and the scrum (Milburn, 1990), potentially resulting in an increase in the incidence and severity of injury.

Rugby union is a collision sport with a high incidence of injury and the highest incidence of time-loss injury compared with other sports among adolescent athletes (Bleakley et al., 2011). The comparatively high risk for injury in rugby may be influenced the many changes and variance in size and function that accompany the pubertal growth spurt (Caine et al., 2014). Boys who mature in advance of their peers benefit from an earlier and often more intense growth spurt, resulting in marked advantages in size, strength, speed, power, and momentum. Maturity associated differences in athleticism emerge from approximately 11 years of age and are maintained through late adolescence, before attenuating in early-to-middle adulthood. Consequently, major differences have been observed in body weight and body composition (Gavarry et al., 2018), maturity (Nutton et al., 2012), and physical qualities of strength and power (McCunn et al., 2017; Perroni et al., 2018) between young players in the same age category during puberty.

In rugby union, serious attention is being paid to minimize and control the risk of injury and promote competitive equity in youth categories. Thus, classifying young rugby players by age is a matter of debate in some rugby federations with a growing awareness to evolve the age-grading model (Patton et al., 2016). Further to these concerns, there is an argument that categories based on size rather than age may help address the competitive inequity associated with maturity-associated variance in size and function, and present early and late developing players more optimal and developmentally appropriate challenges and learning opportunities. Several authors proposed to cross the age-based grading reference with other methods such as the skill-based grading (McCoy et al., 1984), the body mass–based grading used by the New Zealand Rugby Union (World Rugby, 2016), the biological-maturation-based grading (Cumming et al., 2017), or other holistic and hybrid strategies (Tucker et al., 2016). It should be noted that these grouping strategies need not serve as a replacement for age group competition but exist as an additional competitive format that is part of a diversified games programs. Anthropometric characteristics, such as weight, can serve as objective, convenient, and potential dispensation criteria among the youth rugby players (Patton et al., 2016). Although adding a weight restriction is expected to reduce variability, to date, no study has been conducted to propose a weight-grading model. Moreover, knowing that for a given body mass index (BMI) corresponds to different contributions of body fat mass and fat-free mass and that different correlations exist between BMI and indexes of body composition during puberty (Gavarry et al., 2018), there is a need to include body composition and BMI status in the analysis of the weight-grading model. Debating the question of whether rugby should be played according to weight categories in young players, Lambert et al. (2010) has also been suggested that it is unrealistic to categorize only by weight without taking into account body composition parameters.

In light of the preceding discussion, the aim of this study was to compare the anthropometric characteristics of a weight-grading model with the age-grading model, taking into account BMI status and the contribution of body fat mass index (BFMI) and fat-free mass index (FFMI) to the changes in BMI. This study also aimed to identify standardized morphotypes plotted on Hattori’s body composition chart (Hattori et al., 1997) across different weight categories. It was hypothesized that the weight-grading model decreases body weight, body fat mass, and fat-free mass variations between young players.

## Materials and Methods

### Participants and Data Collection

One thousand young male rugby union players from a population of 4,442 aged 9 to 14 years from Provence-Côte d’Azur rugby county (France) were included in a cross-sectional study. From these children, 738 volunteered have to be integrated into this study and all the players were attached to clubs. This study was conducted between September 2012 and May 2013. Informed consent was obtained from child and parent or legal guardian before each child’s participation. The study was approved by the Ethics Committee of the University of Toulon and was conducted in accordance with the Declaration of Helsinki.

### Determination of BMI

Height was measured using a portable stadiometer (Leicester high measure; Tanita, UK). Body mass was measured using an electronic weighing scale (SECA 920, class 3, Germany). Both were measured, respectively, to the nearest 0.1 cm and 0.1 kg. The participants were dressed lightly without shoes. Then, BMI was calculated as body mass (kg) divided by height squared (m^2^).

### Evaluation of Body Composition

Body composition was assessed by the skinfold thickness method at selected four sites (biceps, triceps, subscapular, and suprailiac) with a Harpenden skinfold caliper (Baty International, England). All skinfolds were measured by the same researcher to eliminate inter-tester variability. The test–retest intraclass coefficients on a random sample of 50 subjects in each age category were greater than 0.99. Three successive measurements of each site were taken. If these three values varied by more than 0.2 mm, two additional measurements were taken. The mean of skinfold measurements at each site was used for statistical analysis. The protocol for precise skinfold location and measurement was carefully followed, according to the standardized procedures and guidelines described by Lohman (1992). According to the equations of Durnin and Rahaman (1967), percent body fat and fat-free mass were determined: body density (BD) = 1.1533 - 0.0643 × log sum of 4 skinfolds (triceps, biceps, subscapular, suprailiac), %BF = (4.95/BD - 4.5) × 100. The Durnin and Rahaman equation has been validated against the DXA method in adolescent athletes aged of 15 years (Fonseca-Junior et al., 2017). Fat mass and fat-free mass were then expressed in kilograms to calculate BFMI (kg m^–2^) and FFMI (kg m^–2^) making it possible to adjust body composition to height (VanItallie et al., 1990), BMI (kg m^–2^) = BFMI (kg m^–2^) + FFMI (kg m^–2^).

### Classification of Overweight and Obesity

The prevalence of overweight and obesity was determined using the IOTF (International Obesity Task Force) criteria (Cole et al., 2000). These criteria have been used in a previous study (Gavarry et al., 2018) to classify young players as normal-weight (NW) and overweight/obese players (OW/OB), across age categories.

### Age-Based Grading

The young rugby players had been graded according to age categories (Uage) set by the French Rugby Federation (FRF) which was in force until 2014: Under 11 years (U11: 9.8 ± 0.7 years, *n* = 246), Under 13 years (U13: 11.6 ± 0.8 years, *n* = 260), and Under 15 years (U15: 14.0 ± 0.6 years, *n* = 230). Age was recorded as a decimal value for each child using their date of birth and the date of testing.

### Body Mass–Based Grading

The young rugby players had been graded according to the weight categories (Wx-y) set by the cut-off limit for the 25th and 75th percentiles: U_11_ (25th: 32.4 kg and 75th: 44.1 kg), U13 (25th: 39.7 kg and 75th: 54.9 kg), and U15 (25th: 61.0 kg and 75th: 79.9 kg). These cut-offs were harmonized to have a continuity without overlap of weight: W30–44.9 (from 30 to 44.9 kg, 10.4 ± 1.1 years, *n* = 268), W45–59.9 (from 45 to 59.9 kg, 11.7 ± 1.5 years, *n* = 194), and W60–79.9 (from 60 to 79.9 kg, 13.6 ± 1.2 years, *n* = 167).

### Time of Practice of Sport

The time spent in practicing sport (school physical activity and rugby training) have been evaluated using a survey during the competitive season: school physical activity (U11: 3.0 ± 0.0, W30–44.9: 3.5 ± 0.5, U13: 4.0 ± 0.0, W45–59.9: 3.7 ± 0.6, U15: 2.9 ± 1.0, and W60–79.9: 3.0 ± 1.0 hours/week) and rugby training (U11: 3.8 ± 0.4, W30–44.9: 3.9 ± 0.5, U13: 4.1 ± 0.6, W45–59.9: 4.3 ± 0.8, U15: 5.2 ± 1.2, and W60–79.9: 4.9 ± 1.2 hours/week).

### Body Composition Chart

To graphically present in two-dimensional chart the different body composition aspects such as FFMI, BFMI, percent body fat mass, and BMI of each grading models in youth rugby union players, Hattori’s (1997) model has been used. FFMI, BFMI, percent body fat mass, and BMI were included in different axis in the same chart, with percent body fat mass = BFMI/(BFMI + FFMI).

### Morphotype Subgroups

From Hattori’s body composition chart, the FFMI and the BFMI were classified into three subgroups, respectively. More precisely, groups located below the mean - 1 SD, between the means ± 1 SD, and above the mean + 1 SD were, respectively, characterized as slender, intermediate, and solid for the FFMI, and lean, intermediate, and adipose for the BFMI.

### Scatter Diagram Comparisons From the Two Grading Models

The scatter diagrams of the BFMI and FFMI for both grading models were circumscribed in bivariate normal ellipses (*p* = 0.95) to clarify patterns of diversification of each index estimated relative to that of the other. These and their centroids were modeled using GNU Octave 5.2. The design of the scatter diagram points and centroids were, respectively, open circles and filled circles for the age-based grading and cross for weight-based grading. Bivariate normal ellipses are shown separately for each corresponding grading: U11 vs. W30–44.9, U13 vs. W45–59.9, and U15 vs. W60–79.9.

### Statistical Analysis

The statistical analysis was performed using Statistica 6.1 (Statsoft, 1984–2003). Parameters studied had a normal distribution as assessed by the Kolmogorov–Smirnov test. The variability of anthropometric characteristics in each group was analyzed by the coefficient variation (CV) = SD/mean × 100. An approximate F test was used to compare CV. A one-way ANOVA was used to compare the values of anthropometric characteristics between groups (U11 vs. W30–44.9, U13 vs. W45–59.9, and U15 vs. W60–79.9). Post hoc comparisons were made using Fisher’s LSD test. The magnitude of effects (i.e., effect size) was calculated using Cohen’s d: small (*d* = 0.2), medium (*d* = 0.5), and large (*d* > 0.8). The statistical analysis of bivariate normal ellipses and centroids was performed using XLSTAT 2020.2.2 (by Addinsoft). The lambda of Wilks procedure was used to compare the difference between the centroids of the two grading model groups from the coordinates of the arithmetic means of BFMI and FFMI. The box procedure was used to compare the difference between the distribution deviates from centroids of BFMI and FFMI. Statistical significance was set at *p* < 0.05.

## Results

### Comparison Between the Age-Based Grading and the Weight-Based Grading Models

The comparison of anthropometric characteristics between the age-grading model and the weight-grading model are presented in Table 1. Due to the cut-off limit for the 25th and 75th percentiles, some players were excluded (under W30–44.9: *n* = 34; above W60–79.9: *n* = 64) in the body mass–based grading model.

**TABLE 1:** Comparison of the anthropometric characteristics between the age-based grading and the body mass–based grading models.

	U11 (*n* = 246)	W30–44.9 (*n* = 268)	U13 (*n* = 260)	W45–59.9 (*n* = 194)	U15 (*n* = 230)	W60–79.9 (*n* = 167)
**Weight (kg)**						
X ± SD	39.0 ± 9.2	37.5 ± 4.0	47.8 ± 11.6	51.8 ± 4.4***	71.8 ± 14.0	67.8 ± 5.6***
Median	37.1	37.5	46.1	51.2	69.3	67.0
Range	[22.7; 87.2]	[30.2; 45.0]	[27.6; 99.4]	[45.0; 59.9]	[43.0; 127.3]	[60.1; 80]
CV (%)	23.7	10.8***	24.3	8.6***	19.5	8.2**
**Height (cm)**						
X ± SD	141.4 ± 7.4	143.5 ± 6.8**	152.6 ± 9.2	154.3 ± 8.1*	168.7 ± 7.8	167.2 ± 8.1
Median	141.0	143.0	152.5	154.4	169.0	168.0
Range	[122.4; 162.0]	[126.4; 163.5]	[133.0; 186.0]	[134.5; 176.0]	[147.0; 190.0]	[138.6; 190.0]
CV (%)	5.2	4.8	6.0	5.3	4.6	4.9
**BMI (kg/m^2^)**						
X ± SD	19.3 ± 3.3	18.3 ± 1.8***	20.3 ± 3.4	21.9 ± 2.5***	25.2 ± 4.2	24.3 ± 2.5**
Median	18.5	18.0	19.9	21.6	18.0	24.4
Range	[14.2; 34.5]	[14.4; 26.5]	[12.8; 34.0]	[16.0; 34.5]	[14.4; 42.0]	[19.0; 31.6]
CV (%)	16.9	9.9***	16.7	11.3***	16.6	10.4**
**BFMI (kg/m^2^)**						
X ± SD	4.4 ± 2.5	3.6 ± 1.3***	4.7 ± 1.9	5.7 ± 2.1***	6.7 ± 2.4	6.3 ± 1.8
Median	3.5	3.3	4.3	5.3	6.5	6.3
Range	[1.0; 16.8]	[1.3; 9.2]	[1.6; 12.2]	[2.2; 16.8]	[1.8; 15.4]	[3.1; 12.1]
CV (%)	57.8	35.7***	40.9	37.4	36.2	28.7**
**FFMI (kg/m^2^)**						
X ± SD	14.9 ± 1.3	14.6 ± 1.0	15.6 ± 1.8	16.1 ± 1.3***	18.5 ± 2.0	17.9 ± 1.3**
Median	14.7	14.5	15.3	16.0	18.1	17.9
Range	[9.9; 19.2]	[9.9; 18.5]	[11.2; 24.4]	[13.5; 24.4]	[12.6; 26.6]	[14.8; 22.3]
CV (%)	8.5	6.9***	11.5	8.1***	10.8	7.3**

Data presented as mean ± SD. One-way ANOVA for weight (*p* < 0.001, η^2^*_*p*_*: 0.67), height (*p* < 0.001, η^2^*_*p*_*: 0.63), BMI (*p* < 0.001, η^2^*_*p*_*: 0.40), BFMI (*p* < 0.001, η^2^*_*p*_*: 0.22), and FFMI (*p* < 0.001, η^2^*_*p*_*: 0.48). Significant differences from previous age model (post hoc comparison Fisher’s LSD): *p < 0.05, **p < 0.01, ***p < 0.001. BFMI, body fat mass index; CV, coefficient of variation; FFMI, fat-free mass index; X ± SD, mean ± standard deviation.

*U11 vs. W30–44.9.* No significant difference was observed for the mean values for weight and FFMI. However, the mean value for height (+2.1 cm/+1.5%, *p* < 0.01, *d* = 0.29) was significantly greater in W30–44.9 than in U11, and conversely the mean values for BMI (−1 kg m^–2^/−5.2%, *p* < 0.001, *d* = −0.37) and BFMI (−0.8 kg m^–2^/−18.2%, *p* < 0.001, *d* = −0.40) were significantly lower. When compared with age groups, the weight-graded strategy resulted in a significant reduction (*p* < 0.001) in the coefficients of variance for weight, BMI, BFMI, and FFMI, but not height (*p* = 0.21). The magnitude of the statistically significant reductions in variance ranged from small to moderate (percentage reduction: weight = 54.4%, BMI = 41.4%, BFMI = 38.2%, FFMI = 18.8%).

*U13 vs. W45–59.9*. Mean values for weight (+4.0 kg/+8.4%, *p* < 0.001, *d* = 0.42), height (+1.7 cm/+1.1%, *p* < 0.05, *d* = 0.19), BMI (+1.6 kg m^–2^/+7.9%, *p* < 0.001, *d* = 0.51), BFMI (+1.0 kg m^–2^/+21.3%, *p* < 0.001, *d* = 0.48), and FFMI (+0.5 kg m^–2^/+3.2%, *p* < 0.001, *d* = 0.31) were significantly higher in W45–59.9 than in U13. In comparison with age groups, the weight-graded strategy resulted in a significant reduction (*p* < 001) in the coefficients of variation for weight, BMI, and FFMI, but not height (*p* = 0.07) and BFMI (*p* = 0.25). The magnitude of the statistically significant reductions in variance ranged from small to moderate (percentage reduction: weight = 64.6%, BMI = 32.3%, FFMI = 29.6%).

*U15 vs. W60–79.9.* No significant differences were found between the mean values for height and BFMI. However, the mean values for weight (−4.0 kg/−5.6%, *p* < 0.001, *d* = −0.35), BMI (−0.9 kg m^–2^/−3.6%, *p* < 0.01, *d* = −0.25), FFMI (−0.6 kg.m^–2^/-3.2%, *p* < 0.01, *d* = −0.34) were significantly higher in W60–79.9 than in U15. In comparison with age groups, the weight-graded strategy resulted in a significant reduction (*p* < 0.01) in the coefficients of variance for weight, BMI, BFMI, and FFMI, but not height (*p* = 0.38). The magnitude of the statistically significant reductions in variance ranged from small to moderate (percentage reduction: weight = 57.9%, BMI = 37.3%, BFMI = 20.7%, FFMI = 32.4%).

### Down- and Upgraded Players From Age Category to Weight Category

Table 2 shows the percentage of young rugby players who moved from age category to weight category. The downgraded U11 and the upgraded U15 players have not been taken into account. Thus, after weight grading, 60% of U11, 55.7% of U13, and 77.8% of U15 remained in the weight category which corresponds to their age group. However, 39.6% of U13 and very few U15 (0.4%) downgraded in W30–44.9, and 20.1% of U15 downgraded in W45–59.9. Also, 24.2% of U11 upgraded in W45–59.9, and 4.2% of U11 and 18% of U13 upgraded in W60–79.9.

**TABLE 2:** Distribution of age-based grading into weight-based grading.

	U11		U13		U15	
	n	%	n	%	n	%
**W30–44.9**	161	60	106	39.6	1	0.4
**W45–59.9**	47	24.2	108	55.7	39	20.1
**W60–79.9**	7	4.2	30	18	130	77.8

**FIGURE 1:**
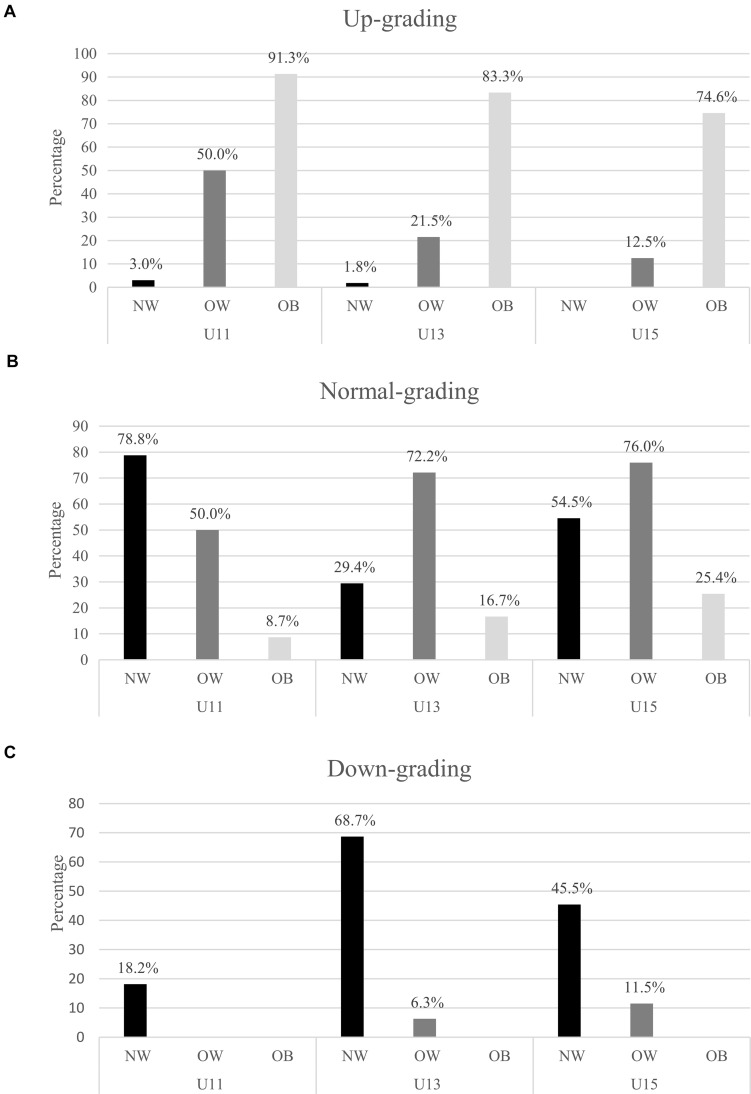
**(A–C)** Change of category from age model to weight model expressed by the percentage of normal, up-, and downgrading depending of BMI status (NW, OW, and OB). NW, normal weight; OB, obese; OW, overweight.

**FIGURE 2:**
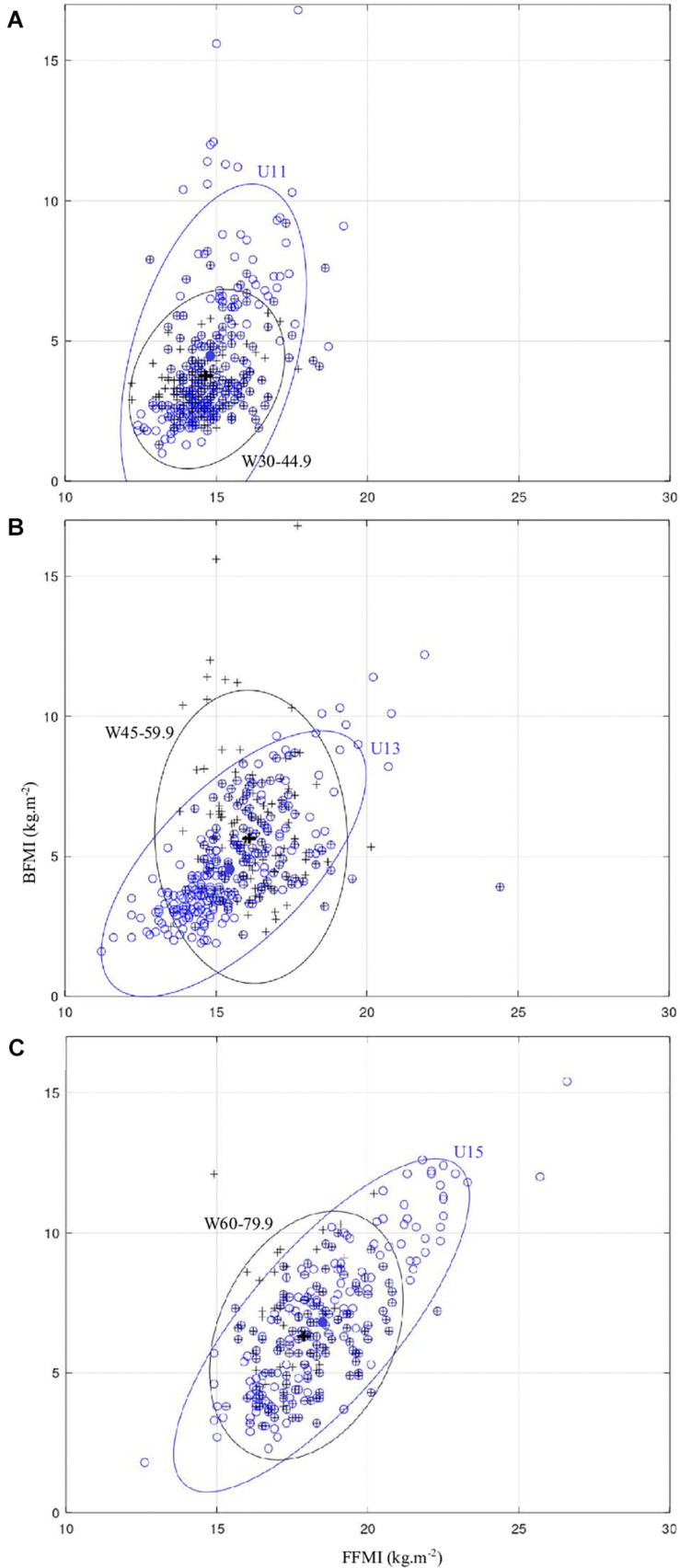
**(A–C)** Scatter diagram of the BFMI and FFMI for age-based grading (open circle) and weight-based grading (cross). Bivariate normal ellipses (blue and black for age- and weight-based grading, respectively) and centroids (filled circle and cross for age- and weight-based grading, respectively). Ellipses, *p* = 0.95. BFMI, body fat mass index; FFMI, fat-free mass index.

The change of category from age grading to weight grading (up- and downgrading) depending on BMI status is presented in Figure 1. In normal-weight players, 18.2% of U11 and 6.7% of U13 were downgraded to under W30–44.9. Moreover, 62% of U13 were downgraded to W30–44.9. On the contrary, only 1.5% of U15 were downgraded to W30–44.9 and 43.9% of them were downgraded to W45–59.9. A very low percentage of U11 (3%) and U13 (1.8%) were upgraded to W45–59.9 and W60–79.9, respectively. In overweight players, a low percentage of U13 (6.3%) and U15 (11.5%) were downgraded to W30–44.9 and W45–59.9, respectively. Half of U11 and 21.5% of U13 were upgraded to W45–59.9 and W60–79.9, respectively. Also, 12.5% of U15 were upgraded to more than W60–79.9. In players categorized as obese, 30.4% of U11 and 55.6% of U13 were upgraded to W60–79.9. Moreover, 4.3% of U11, 27.8% of U13, and 74.6% of U15 were upgraded to more than W60–79.9. Figure 2 shows the comparison of scatter plot of BFMI and FFMI between age grading and weight grading. Although the ellipses are overlapped, their shapes are specific for the type of grading. A flattening of ellipses was observed in each weight category resulting in a decrease of relationships between BFMI and FFMI. More precisely, FFMI was moderately or strongly associated with BFMI in the age model (U11: *r* = 0.80, *p* < 0.001; U13: *r* = 0.66, *p* < 0.001; U15: *r* = 0.77, *p* < 0.001). In contrast, there was no significant correlation between BFMI and FFMI in W45–59.9, and associations between these constructs were weak in W30–44.9 (*r* = 0.25, *p* < 0.001) and W60–79.9 (*r* = 0.29, *p* < 0.001). Lambda of Wilks procedure indicated a significant grading difference between the centroids (Table 1) of BFMI and FFMI (U11 vs. W30–44.9: *p* < 0.0001, *F* = 9.13, λ = 0.97; U13 vs. W45–59.9: *p* < 0.0001, *F* = 14.26, λ = 0.94; U15 vs. W60–79.9: *p* < 0.02, *F* = 3.94, λ = 0.98). Box procedure also indicated a significant grading difference between the distribution deviates from centroids of BFMI and FFMI (U11 vs. W30–44.9: *p* < 0.0001, *F* = 36.86, *M* = 111.05; U13 vs. W45–59.9_:_ *p* < 0.0001, *F* = 32.43, *M* = 97.76; U15 vs. W60–79.9: *p* < 0.0001, *F* = 19.32, *M* = 58.28).

### Morphotype Subgroups From Hattori’s Body Composition Chart in the Weight-Grading Model

**FIGURE 3:**
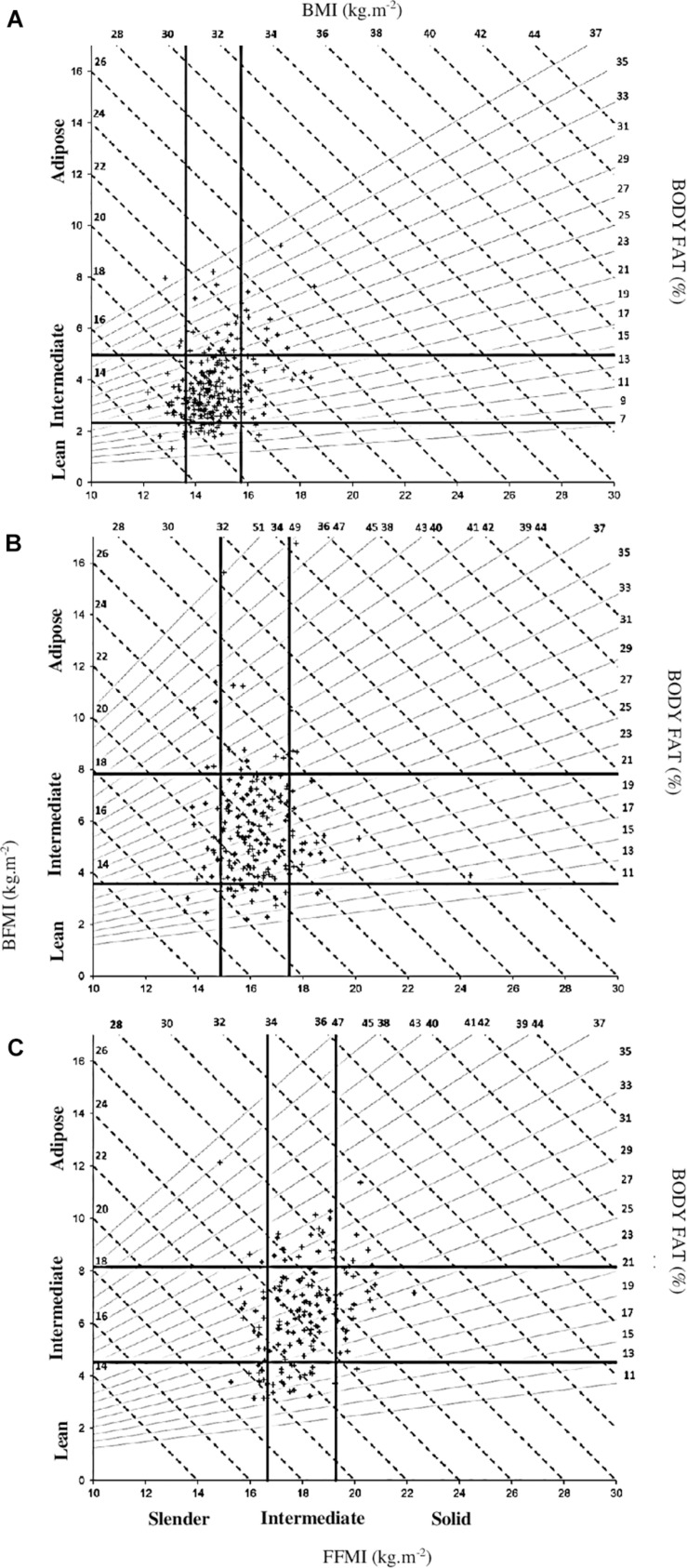
**(A–C)** Distribution of young male rugby players for weight grading model into morphotype subgroups from Hattori’s body composition chart. BFMI, body fat mass index; FFMI, fat-free mass index.

Figure 3 shows the different morphotype subgroups from the body composition classification in the weight-grading model. For FFMI, slender players were located below of 13.6, 14.9, and 16.6 kg m^–2^; intermediate players between 13.6, 14.9, and 16.6 kg m^–2^ and 15.7, 17.5, and 19.3 kg m^–2^; and solid players above 15.7, 17.5, and 19.3 kg m^–2^ in W30–44.9, W45–59.9, and W60–79.9, respectively. For BFMI, lean players were located below 2.3, 3.6, and 4.5 kg m^–2^; intermediate players between 2.3, 3.6, 4.5 kg m^–2^ and 4.9, 7.8, 8.1 kg m^–2^; and adipose players above 4.9, 7.8, and 8.1 kg m^–2^ in W30–44.9, W45–59.9, and W60–79.9, respectively.

Four W30–44.9 (three U11 and one U13), six W45–59.9 (six U11), and three W60–79.9 (two U11 and one U13) had an adipo-slender morphotype. Fourteen W30–44.9 (ten U11 and four U13), four W45–59.9 (two U11, one U13, and one U15), and five W60–79.9 (one U13 and four U15) had an adipo-solid morphotype. Five W30–44.9 (four U11 and one U15), five W45–59.9 (one U11, three U13, and one U15), and twelve W60–79.9 (one U13 and eleven U15) players had the lean-slender morphotype. Two W30–44.9 (two U11), one W45–59.9 (U13), and one W60–79.9 (U15) had the lean-solid morphotype.

## Discussion

This investigation examined variance in the body size and composition of schoolboy rugby (15-a-side rugby) players across the age- and weight-grading grouping models. A number of statistically significant differences in the mean values for body size and compositions were observed between the weight-based and respective age groups. The magnitudes of these differences were, however, comparatively small, suggesting limited impact of the weight grading on the central tendencies for body size and/or composition. Differences were observed, however, in the degrees of variance for body size and composition across the age group and weight-graded groups, with the exception of height. As expected, the weight-graded strategy consistently reduced variance in weight, BMI, and BFMI. Accordingly, the weight-grading strategy appears effective in terms of limiting mismatches in these variables among schoolboy rugby players. The results of the bivariate normal ellipses indicated a decrease of the dispersion of FFMI/BFMI between young players after grading by weight. Moreover, around 56% to 78% of young players are in a weight category that would correspond to their age category, which means that the identity of the category has been in part preserved. However, atypicalities remained (10%), especially concerning extreme morphotypes (adipo-slender, adipo-solid, lean-slender, lean-solid), and have been pointed out in the weight model. The originality of our study lies in the methods chosen to group young rugby players from percentiles scores of weight and in the identification of two-dimensional and standardized morphotypes plotted on Hattori’s body composition chart.

The introduction of weight categories in young rugby players, particularly during puberty, has received particular attention from some rugby union federations like Australia, New Zealand, or independent schools (Patton et al., 2016). A particular concern with respect to age groups is that marked disparities in body size within the age category model may result in an increased risk for injury among smaller and/or later maturing players (Tucker et al., 2016). Although there is currently no evidence to suggest that smaller or later maturing players are at greater risk for injury, there is limited evidence to suggest that this is not the case. Although one study has indicated that larger players were more susceptible to injury incidence (Archbold et al., 2017), this observation may result from larger players dominating gameplay and, thus, having greater exposure for injury. Smaller or later maturing players could participate less actively in contact events.

Another concern pertaining to size disparities in junior rugby is competitive equity. By nature of the superior size, larger and/or more physically mature players are more likely to succeed and be represented on select junior rugby programs (Howard et al., 2016). Although greater size may afford an initial advantage, it may detrimentally impact the long-term development of these players if they rely on their physical advantages at the expense of their technical, tactical, and psychological development (McIntosh et al., 2003). A selection bias toward larger and/or more physically mature players may also result in the deselection and/or loss of many talented smaller and/or later maturing players (Patton et al., 2016). It should be noted that size is only one predictor of performance and selection in junior rugby and that individual differences in biological maturation may also contribute to performance and selection biases. Indeed, research in judo suggests that maturation serves as a stronger predictor of upper body strength than both size and experience in junior athletes (Detanico et al., 2020).

The Weight Consideration Guideline produced by World Rugby (2016) identifies age groups as the most efficient strategy for matching players on the basis of cognitive, psychosocial, and physical development. From a pragmatic perspective, it can also be argued that age group systems are the easiest to implement and regulate. That said, World Rugby do not exclude the consideration of the weight-grading model. The age-group model is currently used by all nations and classification generally involves a single (England or Australia) or 2-year age band, as in France. The consideration of the weight-grading model is particularly relevant in France due to the application of bi-annual age groups. In most nations, regulations exist to permit the combination of players of different age for the purposes of training and/or playing up or down and age group. In the present study, based on a classification integrating bi-annual age groups, marked differences were observed between the heaviest and lightest players within the same age category (U11: 64.5; U13: 71.8; U15: 84.3 kg). Similarly, but at the same age (15 years), Nutton et al. (2012) observed a large weight variation in Scottish schoolboy rugby players (weight range: 46–127.1 kg). This makes it clear that annual-age grading does not reduce weight variability. These large differences raise the obvious question whether the weight-grading model would allow to reduce this large disparity between young players. According to our data, mean weight differed significantly between U13 versus W45–59.9 and U15 versus W60–79.9. However, coefficient of variation for this variable was significantly lower in the weight model compared with the age model (from −11.3 to −15.7 points) suggesting a reduction of dispersion in the weight model. In addition, we observed a very high reduction of the range of weight between the two models (from −49.7 to −64.9 kg). In line with this discussion, it is reasonable to suggest that smaller players should perceive a lower apprehension and a greater motivation to engage in physical contacts in the weight model because of the facilitation of playing with similar-sized players. This could lead to an improvement of tackle and ruck technical proficiency.

Various methods of dispensation involving body-mass criteria are proposed depending on statistical criteria such as percentile, SD, and/or the reference population (Patton et al., 2016). In the present study, we used cut-off limit of the 25th and 75th percentiles as a criterion for changing category, as the Sydney Junior Rugby method, 2011. Unlike this latter method, our analysis was based on our study population of schoolboy rugby players, more relevant in our opinion than the general population with the aim of dispensation. In fact, Sydney Junior Rugby method may underestimate anthropometric characteristics of the young rugby players’ population compared with a normative population (Krause et al., 2015). In this context, Patton et al. (2016) suggested to measure body mass of young rugby players upon pre-season registration to establish updated data between seasons. This allows to overcome morphological changes that occurred over time in this population (Sedeaud et al., 2014). Thus, our weight-grading method proposed a dispensation inside a group belonging to a minimum and maximum weight value under and above which the young players could receive a down- or upgrading, respectively. Interestingly, the mean of chronological age for weight groups was similar to that of age groups (W30–44.9 vs. U11: +0.6; W45–59.9 vs. U13: +0.1; W60–79.9 vs. U15: −0.4 years) but with greater SD. In addition, we can consider that a large number of U11 and U15 were in a weight category that would correspond to their age category. This finding indicated that the age category in terms of weight has been preserved for these young players with the weight model. However, this was not true for U13 for which about 40% of them were downgraded in the W30–44.9 category (Table 2) with a majority of normal-weight players (around 95%). In the present study, upgraded players were mainly obese players independent of age category. This is linked to the fact that majority of obese rugby players (from 66 to 100%) were in the highest tertiles for BFMI and FFMI in the age model. In addition, a dependence between BFMI and FFMI has been pointed out for the age model as shown by moderate and strong correlations between these two components. This means that the difference in BMI was due to fat mass and fat-free mass particularly in U13 and U15 (Gavarry et al., 2018). In contrast, there was no relationship between BFMI and FFMI in each weight category. Although many authors reported an important prevalence of obesity in the young rugby player population, we have observed in the present study a decrease of the contribution of BFMI in the excess BMI. Considering the 75th percentile as the threshold of excess BMI in our study, we found that 78% of excess BMI were explained by fat mass among obese U11. Similar results (75%) have been reported among obese children (Webster et al., 1984; Wells et al., 2006). Lower values were found among the obese U13 (59%) and U15 (54%). Thus, FFMI plays an increasingly important role in the contribution of excess BMI in these obese players with increasing age underlying the need to measure body composition from 11 to 12 years. In line with this result, Krause et al. (2015) showed that the dogma of bigger, faster, and powerful characteristics occur simultaneously in adolescent rugby players was not obvious, and only used body mass criteria for grading would be insufficient.

Our weight category model presents several advantages. First, our results pointed out a decrease of the dispersion in most of anthropometric characteristics for the weight-grading model compared with the age-grading model. The bivariate normal ellipses showed graphically a decrease of dispersion in each weight category (Figure 2). It was characterized by significant differences in centroids and shapes of the ellipses which means that body composition of young rugby players was different in the weight-grading model. It was further supported by a reduction of coefficients of variation for BFMI and FFMI. Second, young players of different age categories were mixed, and consequently taking into account the level of growth by weight and height. It is well known that the rates of growth varied considerably among children between the ages of 12 and 15, and late maturers can start puberty around the age of 13 or 14 years. It is highly likely that these young players were late maturing boys (means weight: 39.2 kg and height: 147.6 cm) and they could receive less attention by coaches to be selected in elite teams with the age-grading model (Armstrong and Welsman, 2005). In contrast, the use of the weight-grading model could help avoid the influence of biological and physical factors in this context. Lastly, knowing that more than half of young American football players classified in advanced maturity were obese in the study of Malina et al. (2007), it could be reasonable to think that obese players of a young age category will find themselves with players with similar maturation level in a high weight category. However, although the dispersion decreased, there are still important differences between the highest and the lowest BFMI (7.9, 14.6, and 9.0 kg m^–2^) and FFMI (8.6, 10.9, and 7.5 kg m^–2^) in the same weight category. Expressed in absolute value, these differences represent 15.2, 22.2, and 28.2 kg for body fat mass and 16.9, 26.7, and 29.7 kg for fat-free mass.

Although a weight-grading strategy may limit variance in size among players, limitations of the weight-grading model should be considered. As has been pointed out in this study, it is necessary to include an evaluation of body composition due to different combinations of body fat mass and fat-free mass for a same weight. In addition, obese players with excess body fat upgraded in a weight category of non-obese older players with similar weight or BMI, but with higher muscle mass and therefore with more powerful and strength. Thus, large variability in body composition can persist. Moreover, category changes can lead to a decrease in self-esteem. The weight model requires to have enough young players to make a team and to organize rugby tournaments according to the weight categories. Psychological, cognitive, and skill development representing important factors of performance were not taken into account in this model and should be also considered (Lambert et al., 2010). As in many weight-category sports, young players could use dangerous nutritional and doping strategies to change weight and body composition. An important imbalance between energy expenditure and energy intake could cause the loss of muscle mass in growing players and weak bones particularly in skeletally immature players, affecting negatively athletic performance and health (Carl et al., 2017). Lastly, maintaining “the spirit of the game” with promotes different body size for the various playing positions is essential. The weight-grading model moves away from this.

Knowing the limits of the weight-grading model, some authors suggested to add anthropometric measures in order to provide objective grading criteria (Lambert et al., 2010; Till et al., 2011; Patton et al., 2016). Thus, there is a need to find a model which could take into account body composition status. In the present study, the use of the two components of BMI (BFMI and FFMI) in the same chart makes it possible to assess whether BMI differences between weight categories are linked to the variations of fat mass or fat-free mass. Although maturation level has not been determined in our population, the use of height normalized for body fat mass and fat-free mass allowed to consider a number of attributes associated with variance in maturation (Wells, 2001). We proposed to use Hattori’s body composition chart including BFMI, FFMI, BMI, and %FM. Several authors promoted BFMI rather than BMI to assess the childhood obesity due to higher sensibility (Schutz et al., 2002; Okorodudu et al., 2010). In the present study, Hattori’s body composition chart pointed out the low ability of BMI to successfully screen body composition of children which corroborates the low sensibility of BMI to detect excess adiposity among the children and adolescents (Javed et al., 2015). According to Freedman et al. (2005), the accuracy of BMI to assess the adiposity varied regarding the level of fatness and the variability of BFMI was two times greater than FFMI. In our study, a great variability of fat mass was observed. For instance, in obese players in U11 with a BMI between 26 and 28 kg m^–2^, fat mass varied from 29 to 45%. In normal players in W30–44.9 with a BMI between 18 and 22 kg m^–2^, fat mass varied from 12 to 29%. Similar observations were observed for each of the age or weight categories. Hattori’s body composition chart reflects the relevant utility to detect a minority of young players with atypical statures. Indeed, this chart allows to distinguish different morphotype subgroups from body composition classifications (thresholds: X ± 1SD) as proposed by several authors (Hattori et al., 1997; Schutz et al., 2002). In the present study, we used the Hattori model terminology to characterize morphotype subgroups. The extreme statures (adipo-slender, adipo-solid, lean-slender, and lean-solid) were accurately identified in Figure 3 and represent 9.3%, 8.2%, and 12.6% in W30–44.9, W45–59.9, and W60–79.9, respectively.

This body composition chart model proposed in the current study has several practical applications. First, an interesting follow-up to this study would be to look at the risk of injury during physical collision depending on morphotypes and body composition of young rugby union players. Another perspective would be to use our model to facilitate a better identification of the overweight among young players. In this way, the contribution of body fat mass and fat-free mass to changes in body composition could be distinguished across age categories in young rugby players classified as obese, overweight, and normal weight by BMI. Moreover, it would be interesting to identify young players with low muscle mass. So, nutritional recommendations, training strategies, and specialization in playing position should be proposed according to the body composition criteria for a prophylactic or performance improvement. That is why regularly monitoring body composition from Hattori’s body composition chart could facilitate the determination of a low muscle mass with or without excess fat mass (Schutz et al., 2002). Many authors have suggested that during the adolescence, different types and regular exercises like jumping activity increased fat-free mass and decreased fat mass (Weeks and Beck, 2012). Finally, there is a need to elaborate reference values and to establish specific cut-off values of FFMI and BFMI from a health and safety perspective of young rugby players. Early prevention of the potential risk for developing cardiovascular disease or to diagnose the predisposition of adolescents to obesity in adulthood through the BFMI and FFMI has to be encouraged.

In conclusion, this study has shown that the weight-grading model allowed to reduce the dispersion of FFMI/BFMI between young players while preserving the identity of age category. The effect of weight grading on player distribution is highly dependent on the method and statistical tools used to create the weight categories. Despite the classification by weight, there was still a lot of variation in body composition. Furthermore, the fact that upgraded players were mainly obese players and that much of excess BMI were explained by fat mass in younger players contributed to maintain body composition mismatches between players in the same weight category. There is a need to include the evaluation of body composition using BFMI and FFMI chart analysis to identify morphotypes and atypicalities and to design individual intervention for fat loss or muscle gain. Further research is needed to improve the weight-grading model taking into account psycho-social dimensions and maturity status.

## Data Availability Statement

The raw data supporting the conclusions of this article will be made available by the authors, without undue reservation.

## Ethics Statement

The studies involving human participants were reviewed and approved by TOULON UNIVERSITY. Written informed consent to participate in this study was provided by the participants’ legal guardian/next of kin.

## Author Contributions

GL, OG, and JP conceived and designed the study. PP, JG, and J-JR assisted with the technical and medical aspects of the protocol, recruited all participants, and involved in the acquisition of data. GL, OG, SC, and PP analyzed the data. GL, OG, and SC performed the statistical analysis. MB provided the mathematic model. GL, OG, PD, and SC drafted the article while MB, JG, and JP revised it critically for important intellectual content. All authors gave final approval of the version to be published.

## Conflict of Interest

The authors declare that the research was conducted in the absence of any commercial or financial relationships that could be construed as a potential conflict of interest.
